# 

**Published:** 1897-06

**Authors:** 


### BOOKS RECEIVED.

Surgical Hints for the Surgeon and General Practitioner. By Howard Lilienthal, M. D., Assistant Attending Surgeon to Mt. Sinai Hospital, New York City. Price, 25 cents. New York: International Journal of Surgery Company. 1897.

A Handbook of Medical Climatology, Embodying its Principles and Therapeutic Application, with Scientific Data of the Chief Health Resorts of the World. By S. Edwin Solly, M. D., M. R. C. S., late President of the American Climatological Association. In one 8vo volume of 470 pages, with engravings and colored plates. Cloth, $4. Philadelphia and New York: Lea Brothers & Co., Publishers. 1897.

Manual of Static Electricity in X-Ray and Therapeutic Uses. By S. H. Monell, M. D., Founder and Chief Instructor of the Brooklyn Post-Graduate School of Clinical Electro-Therapeutics and Röntgen Photography; Fellow of the New York Academy of Medicine. Octavo, 614 pages, cloth, gilt. Illustrated. Price, $5 net; postage, 35 cents. New York: William Beverley Harison, Publisher, 3 and 5 W. 18th street. 1897.

Twenty-second Annual Report of the State Board of Health of the State of Michigan for the Fiscal Year ending June 30, 1894. Henry B. Baker, M. D., Secretary. Lansing: Robert Smith & Co., State Printers and Binders. 1896.

Syringomyelia. An Essay to which was Awarded the Alvarenga Prize of the College of Physicians of Philadelphia, for the Year 1895. By Guy Hinsdale, A. M., M. D., Fellow of the College of Physicians of Philadelphia, etc. Philadelphia: P. Blakiston Son & Co. 1897.

Transactions of the American Microscopical Society. Nineteenth Annual Meeting, held at Carnegie Library, Pittsburg, Pa., August 18, 19 and 20, 1896. William C. Krauss, M. D., Secretary. Buffalo: A. T. Brown Printing House. 1897.

Transactions of the Medical Society of the State of North Carolina. Forty-third Annual Meeting, held at Winston-Salem, N. C., May 12, 13 and 14, 1896. Wilmington: LeGwin Bros., Printers and Binders. 1896.

The Liver of Dyspeptics and Particularly the Cirrhosis Produced by Auto-Intoxication of Gastro-Intestinal Origin. By Dr. Emile Boix, of Paris. Authorised translation from the latest French edition by Paul Richard Brown, M. D., Major and Surgeon U. S. Army. New York: G. P. Putnam's Sons. 1897.

Transactions of the Medical Association of Central New York. Twenty-ninth Annual Meeting, held at Rochester, N. Y., October 20, 1896. William C. Krauss, M. D., Editor. BUFFALO MEDICAL JOURNAL Print. 1897.

